# Antiviral Effect of Isoquercitrin against Influenza A Viral Infection via Modulating Hemagglutinin and Neuraminidase

**DOI:** 10.3390/ijms232113112

**Published:** 2022-10-28

**Authors:** Won-Kyung Cho, Myong-Min Lee, Jin Yeul Ma

**Affiliations:** Korean Medicine (KM) Application Center, Korea Institute of Oriental Medicine, 70 Chemdanro, Dong-gu, Daegu 41062, Korea

**Keywords:** isoquercitrin (IQC), influenza A virus (IAV), neuraminidase (NA), hemagglutinin (HA), virucidal effect

## Abstract

Isoquercitrin (IQC) is a component abundantly present in many plants and is known to have an anti-viral effect against various viruses. In this study, we demonstrate that IQC exhibits strong anti-influenza A virus infection, and its effect is closely related to the suppression of hemagglutinin (HA) and neuraminidase (NA) activities. We used green fluorescent protein-tagged Influenza A/PR/8/34 (H1N1), A/PR/8/34 (H1N1), and HBPV-VR-32 (H3N2) to evaluate the anti-IAV effect of IQC. The fluorescence microscopy and fluorescence-activated cell sorting analysis showed that IQC significantly decreases the levels of GFP expressed by IAV infection, dose-dependently. Consistent with that, IQC inhibited cytopathic effects by H1N1 or H3N2 IAV infection. Immunofluorescence analysis confirmed that IQC represses the IAV protein expression. Time-of-addition assay showed that IQC inhibits viral attachment and entry and exerts a strong virucidal effect during IAV infection. Hemagglutination assay confirmed that IQC affects IAV HA. Further, IQC potently reduced the NA activities of H1N1 and H3N2 IAV. Collectively, IQC prevents IAV infection at multi-stages via virucidal effects, inhibiting attachment, entry and viral release. Our results indicate that IQC could be developed as a potent antiviral drug to protect against influenza viral infection.

## 1. Introduction

Influenza viruses, a family of orthomyxoviridae with segmented RNA genome [1], produce respiratory and systemic symptoms such as cough, sore throat, fever, headache, muscular pain, and pneumonia and have a serious impact, even including death, in high-risk groups [2]. Influenza A virus (IAV) is the main cause of seasonal flu in humans every year. New IAV variants with different hemagglutinin (HA) and/or neuraminidase (NA) are generated due to the frequent antigenic drift and antigen point mutation during the replication of the RNA gene [1,3]. It is impossible to develop the perfect vaccine to protect against new variants in advance. As an antiviral agent, M2 protein inhibitors such as rimantadine and amantadine were used in clinical practice. However, it does not protect against infection from the influenza B virus and has a tolerance to the influenza A virus [4]. Neuraminidase inhibitors such as zanamivir, oseltamivir, peramivir, and baloxavir have been used; however, several reports demonstrated the side effects and drug resistance [5,6] of these inhibitors. Many studies are underway to find novel antiviral agents against influenza A virus infection from natural products or single compound libraries with virtual screening [7,8,9,10].

Isoquercitrin (IQC) is a flavonoid, which is rich in various plants such as *Mangifera indica*, *Rheum nobile*, *Annona squamosa*, *Camellia sinensis*, and *Vestia foetida*. It is also called isoquercetin, isotrifoliin, or quercetin 3-glucoside. IQC has been reported to have efficacy against various diseases and has anti-cancer [11], anti-fatigue [12], anti-diabetes [13,14,15], and neuroprotective effects [16,17,18]. IQC is also known to have a broad spectrum of antiviral activities. Several reports have demonstrated that IQC has antiviral effects against the Zika virus [19,20], herpes virus infection [21,22], Ebola virus [23], and Mayaro virus [24]. In addition, Kim Y et al. [25] showed that IQC inhibited influenza A and B viral replication. They reported that the double treatment of IQC and amantadine exerted a synergistic effect on IAV replication. Recently, Kai G et al. [26] reported that quercetin 3-glucoside in *Dianthus superbus* L inhibits IAV infection through the suppression of ROS, acidic vesicular organelle formation, and PB2 protein. In the present study, we first demonstrate that the antiviral effect of IQC against IAV infection is related to the suppression of viral binding and entry and a virucidal effect via the blockage of hemagglutination in the early stages of IAV infection. Further, IQC exhibited inhibitory effects on the neuraminidase of H1N1 and H3N2 IAV.

## 2. Results

### 2.1. Cytotoxicity of Isoquercitrin (IQC)

**Figure 1:**
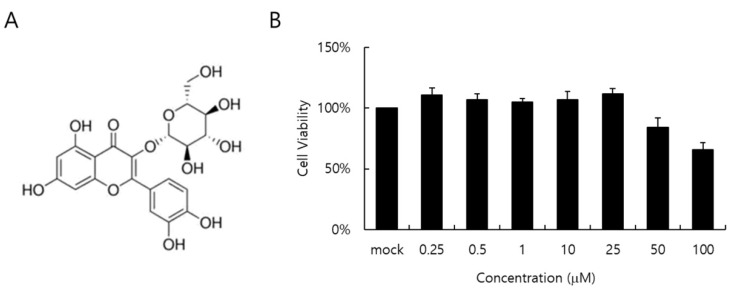
The chemical structure (**A**) and cytotoxicity (**B**) of isoquercitrin (IQC). IQC was treated to the RAW 264.7 cells with indicated concentrations for 24 h at 37 °C. Cytotoxicity was detected using a CCK-8 assay. The data represent the mean ± SD based on three replicates in three different experiments.

The cytotoxicity of IQC to RAW 264.7 cells was determined by a CCK-8 assay. When the cell viability was checked at 24 h post-treatment with IQC at indicated concentrations, IQC did not show a toxic effect on the cell viability up to 25 µM (Figure 1B).

### 2.2. Anti-Influenza Viral Effect of Isoquercitrin

**Figure 2:**
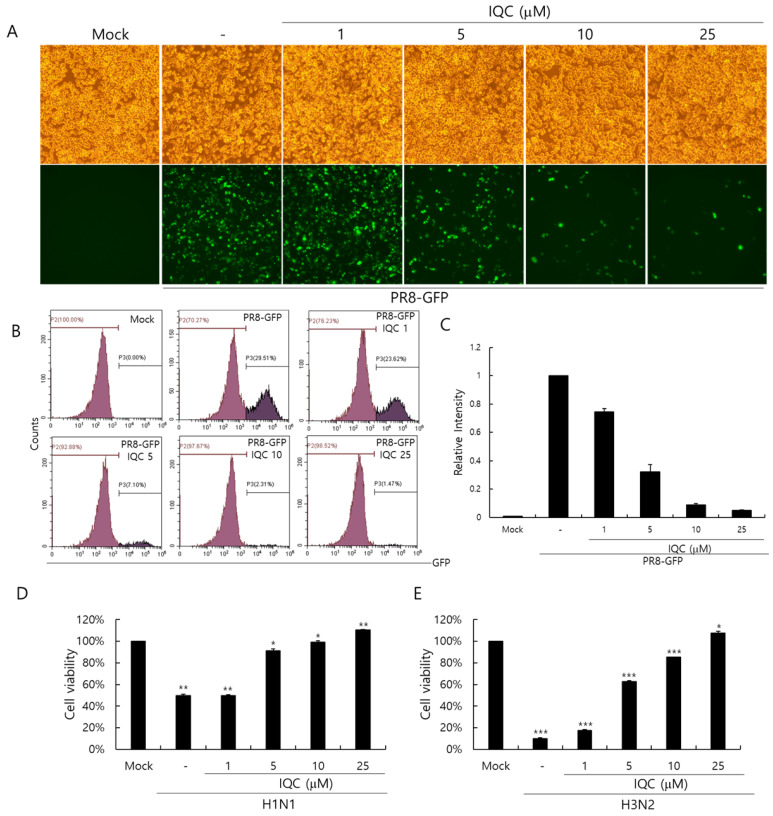
The antiviral effect of isoquercitrin against influenza A viral infection. (A-C) IQC at 0, 1, 5, 10, or 25 μM with 10 MOI PR8-GFP IAV were mixed for 1 h at 4 °C, the mixtures were cotreated to RAW 264.7 cells for 2 h at 37 °C. After washing with PBS, the cells were further incubated for 24 h at 37 °C. (**A**) Brightfield and fluorescence images were obtained using the fluorescence microscope with 200× magnification. (**B**,**C**) The cells were fixed with 4% paraformaldehyde and analyzed by FACS. The levels of GFP expression were depicted as relative intensities compared to the PR8-GFP IAV-infected control. The data represent the mean ± SD based on three independent experiments. (**D**) H1N1 or (**E**) H3N2 IAV at 50 MOI was incubated with IQC at the indicated concentrations 1 h at 4°C and the mixture was added to the cells until cytopathic effect formation at 37 °C. The supernatant was harvested to assess the cell viability using a CCK-8 assay. The data represent the mean ± SD based on three independent experiments. Statistical significance was assessed via an unpaired Student *t*-test. *** *p* < 0.001, ** *p* < 0.005, * *p* < 0.05.

We used the GFP-tagged Influenza A/PR8/34 (PR8-GFP IAV) virus to assess the anti-influenza viral effect. IQC (0, 1, 5, 10, or 25 μM) and PR8-GFP IAV were mixed and placed for 1 h at 4 °C, and were treated to the RAW 264.7 cells for 2 h at 37 °C. The cells were washed with PBS and further incubated for 24 h at 37 °C to express GFP. As shown in Figure 2A, the cells infected by PR8-GFP IAV strongly expressed GFP, but IQC significantly decreased GFP expression dose-dependently. To confirm the inhibitory effect of IQC on the influenza viral infection, we performed an FACS analysis on the cells infected by PR8-GFP IAV in the absence or presence of IQC (Figure 2B). The levels of GFP expression in each group were compared with the IAV-infected control group and the values of each group were depicted as relative intensities (Figure 2C). IQC inhibited the PR8-GFP IAV-induced GFP expression with IC50 of 2.68 ± 0.35 µg/mL. Consistently, IQC potently suppressed GFP expression by PR8-GFP IAV infection. Next, we examined whether IQC could inhibit other types of IAV such as H1N1 and H3N2 IAV. The viability of cells infected with H1N1 or H3N2 IAV was significantly decreased by cytopathic effects. However, IQC dose-dependently suppressed the cytopathic effects by H1N1 and H3N2 IAV infection, as presented in Figure 2D,E. The IC50 values of IQC for H1N1 and H3N2 IAV infection were determined to be 3.52 ± 0.34 and 3.92 ± 0.07 µg/mL, respectively.

### 2.3. Isoquercitrin Inhibits Influenza Viral Protein Expression

**Figure 3:**
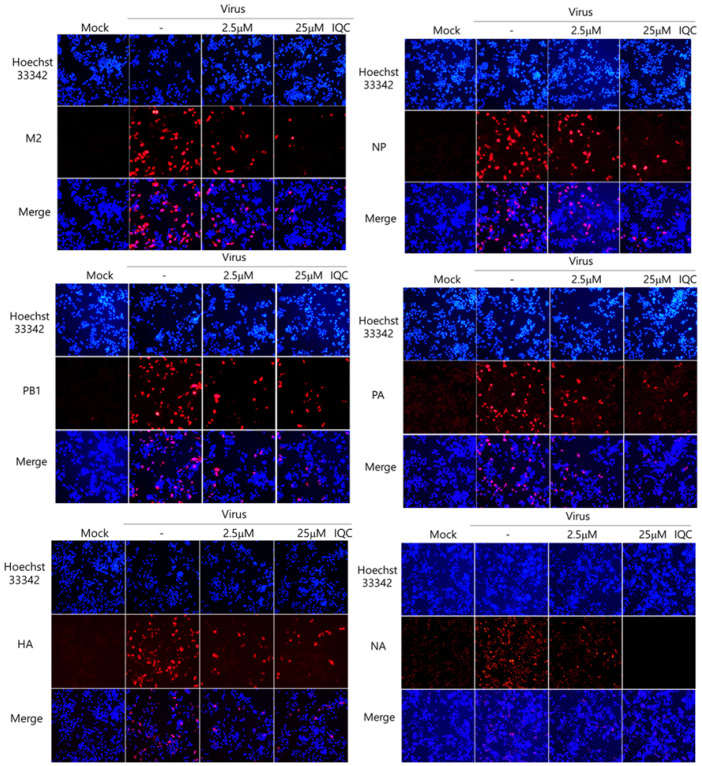
Isoquercitrin significantly decreased influenza A viral protein expression. PR8-GFP IAV at 10 MOI and 0, 2.5 µM, or 25 µM IQC were mixed and cotreated to the RAW 264.7 cells, as described in Figure 2. After fixing with paraformaldehyde at 24 h post-infection, the cells were immuno-stained with specific antibodies against M2, NP, PB1, PA, HA and NA (Red). The nuclei were stained in blue color with Hoechst 33342. The co-localization of viral proteins and nuclei was observed using a fluorescent microscope.

Next, we examined whether IQC could affect IAV protein expression using immunofluorescence analysis. IQC (2.5 μM or 25 μM) and PR8-GFP IAV were mixed for 1 h at 4 °C and cotreated to the RAW 264.7 cells for 2 h at 37 °C. The cells were further incubated for 24 h at 37 °C after washing with PBS and were fixed with absolute methanol and 4% paraformaldehyde. The cells were stained with Alexa 594-tagged antibodies against M2, NP, HA, PA, PB1, and NA viral proteins. The nuclei in the cells were detected by staining with Hoechst 33342. Figure 3 shows that IQC at both 2.5 μM and 25 μM repressed all viral proteins, and 25 μM IQC potently decreased viral proteins. These results indicate that IQC significantly inhibits IAV protein expression dose-dependently.

### 2.4. Effect of Isoquercitrin on IAV Infection at Early Stages

**Figure 4:**
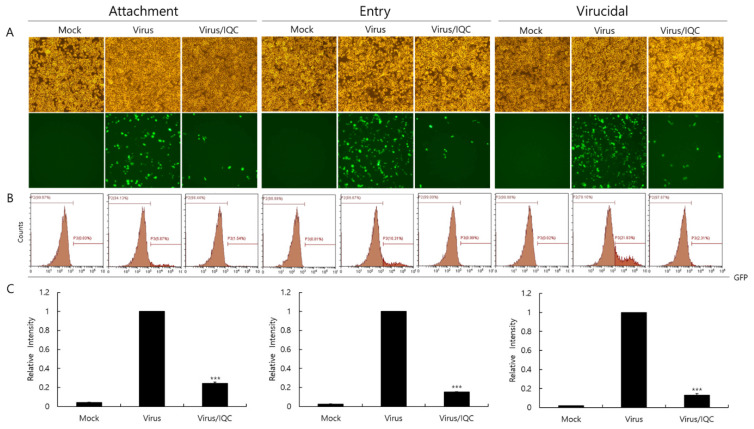
Effect of isoquercitrin on viral attachment, entry, and virucidal stages. (**A**–**C**) For checking the effect of IQC on the attachment stage, RAW 264.7 cells were cotreated with PR8-GFP IAV at 10 MOI and IQC at 25 µM for 30 min at 4 °C. After washing, the cells were incubated for 24 h at 37 °C. To examine the effect of IQC on the entry stage, the cells were infected with 10 MOI PR8-GFP IAV at 4 °C for 30 min, followed by the treatment of 25 µM IQC for 30 min at 37 °C. After washing, the cells were incubated for 24 h at 37 °C. For investigation of the effect of IQC on the virucidal stage, 10 MOI PR8-GFP IAV and 25 µM IQC were mixed and incubated for 30 min at 4 °C. The cells were added with the mixtures and incubated for 30 min at 37 °C. After washing, the cells were incubated for 24 h at 37 °C. (**A**) The cell images were captured using fluorescence microscopy with 200× magnification. (**B**,**C**) The cells were fixed with paraformaldehyde and analyzed using FACS. The levels of GFP expressed in the cells were calculated by comparison with PR8-GFP IAV. The data represent the mean ± SD value based on three independent experiments. Statistical significance was assessed via an unpaired Student *t*-test. *** *p* < 0.0001.

Since IQC significantly inhibited IAV infection, we examined whether IQC affects IAV infection at the early stages. First of all, the RAW 264.7 cells were treated with medium, PR8-GFP IAV or a mixture of PR8-GFP IAV and IQC for 30 min at 4 °C before incubation for 24 h at 37 °C to investigate the effect of IQC on viral binding to the cells. To examine the effect of IQC on the viral entry into the cells, the RAW 264.7 cells were infected with medium or PR8-GFP for 30 min at 4 °C before adding IQC. After washing the cells to remove unbound virus, the cells were incubated with medium or IQC for 30 min at 37 °C, followed by further incubation for 24 h at 37 °C after the removal of IQC. We also checked whether IQC could kill the virus before viral infection into the cells. To evaluate the virucidal effect of IQC, medium, PR8-GFP IAV only, or a mixture of PR8-GFP IAV and IQC were incubated for 30 min at 4 °C. Each mixture was treated to the RAW 264.7 cells for 30 min at 37 °C. After washing, the cells were further incubated for 24 h at 37 °C. Figure 4 shows that IQC potently blocks virus attachment and entry to the cells and, further, it exerted a strong virucidal effect. These results indicate that IQC has a strong anti-IAV effect by inhibiting viral infection at the early stages. 

### 2.5. Effect of Isoquercitrin on Hemagglutination

**Figure 5:**
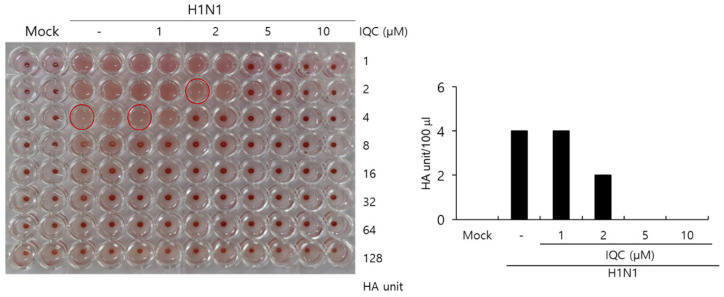
Isoquercitrin inhibits hemagglutination upon IAV infection. (**A**,**B**) H1N1 IAV at 10 MOI and IQC at concentrations of 0, 1, 5, 10, or 25 µM were mixed for 1 h at 4 °C. Each mixture was added to the RAW 264.7 cells for 2 h. After washing with PBS, the cells were further incubated for 24 h. The supernatants of cells were serially 2-fold diluted and mixed with 1% RBC cells to investigate the effect of IQC on hemagglutination by IAV infection.

The HA protein of the influenza virus is crucial for viral attachment to the cells and inducing hemagglutination in red blood cells. Since IQC has a strong inhibitory effect on viral attachment and entry during IAV infection, we examined whether IQC could influence hemagglutination by IAV infection. To address this, H1N1 IAV and IQC at various concentrations were cotreated to the RAW 264.7 cells, as described in Figure 2. At 24 h post-incubation, the supernatants of the medium, virus-only, and the mixtures of IAV and IQC were collected. The supernatants were serially diluted 2-fold and mixed with 1% chicken red blood cells for 1 h at RT. As shown in Figure 5, IQC significantly decreased the HA units of H1N1 IAV. The control virus expressed four HA units in the absence of IQC. However, IQC dose-dependently reduced HA units. HA units at 2 µM of IQC were 2-fold lower than that of the control, and at 5 or 10 µM of IQC completely blocked hemagglutination. This result implies that IQC strongly inhibits influenza HA protein, which is closely related to viral attachment and entry.

### 2.6. Isoquercitrin Suppressed on Neuraminidase Activity

**Figure 6:**
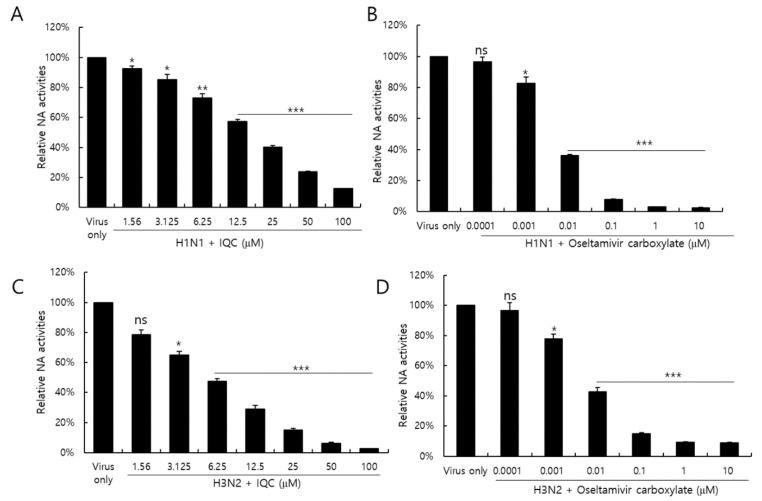
The inhibitory effect of isoquercitrin on H1N1 and H3N2 IAV neuraminidase activities. After mixing H1N1 (**A**) or H3N2 IAV (**C**) with 2 × 10^6^ PFU and IQC at the indicated concentration, each mixture was incubated with 200 µM NA-Fluor Substrate for 1 h for 37 °C. Oseltamivir carboxylate as a positive control was mixed with H1N1 (**B**) or H3N2 (**D**) IAV. After termination with NA-Fluor stop solution, the NA activities were measured by a fluorescent spectrometer with an excitation wavelength of 365 nm and an emission wavelength of 445 nm. The data represent the mean ± SD based on three independent experiments. Statistical significance was assessed via an unpaired Student *t*-test. *** *p* < 0.0005, ** *p* < 0.001, * *p* < 0.005, ns; no significance.

Neuraminidase activity of the influenza virus is essential for the release of IAV progeny from the cells. Neuraminidase inhibitors, such as oseltamivir and zanamivir, are well-known anti-influenza viral drugs. We investigated whether IQC could inhibit the NA activity of IAV. Figure 6 shows that the NA activities of H1N1 and H3N2 IAV were significantly reduced in the presence of IQC, dose-dependently (Figure 6A,C). Oseltamivir carboxylate (OC), used as a positive control, potently repressed NA activity (Figure 6B,D). At a concentration of 25 μM, IQC inhibited H1N1 and H3N2 NA activities by 60% and 80%, comparable with 0.01 μM or 0.1M of OC, respectively. The NA activities of H1N1 and H3N2 IAV were suppressed by IQC with an IC50 value of 17.21 ± 0.28 and 5.91 ± 0.49 µg/mL, respectively. These results indicate that IQC inhibits IAV progeny release from the cells via blocking NA activity at the late stage during IAV infection.

## 3. Discussion

In the previous study, we reported that lotus (*Nelumbo nucifera* Gaertn.) leaves extract exhibits antiviral efficacy by directly inhibiting IAV infection [27]. We also showed that, among the major components present in lotus leaves, isoquercitrin is an effective compound for anti-IAV effects. This study was conducted to address how IQC exerts its inhibitory effect on IAV infection. We examined the effect of IQC on viral replication using PR8-GFP IAV, which expresses GFP with influenza viral gene expression. The fluorescence analysis with microscopy and FACS showed that IQC potently repressed IAV infection dose-dependently (Figure 2A–C). The inhibitory effect of IQC against IAV was confirmed with the suppression of the cytopathic effect caused by H1N1 or H3N2 IAV infection (Figure 2D,E). Consistent with the results, the immunofluorescence staining demonstrated the inhibitory effect of IQC on IAV protein expression (Figure 3). Next, when we conducted a time-of-addition assay to examine whether IQC influences IAV infection at the early stages, we found that IQC prevented the viral attachment and entry to the cells, and further it exerted potent virucidal efficacy (Figure 4). IAV infection is initiated by the binding of the hemagglutinin of IAV to the glycoprotein receptors with sialic acid on the target cells in the early stage upon IAV infection. Natural products, such as *Camellia sinensis* [28], cranberry extract [29], *Eupatorium perfoliatum* [30] *Jatropha curcas* [31], *Isatis indigotica* [32] and compounds containing triterpene and pentacyclic triterpenes structure [33,34,35,36], are known to prevent influenza viral infection by modulation of HA activity and viral attachment. A hemagglutination inhibition assay to investigate whether IQC affects HA activity showed that IQC from 5 µM completely blocked hemagglutination. These results indicate that IQC inhibits the viral binding to the cell membrane through HA modulation during IAV infection. The virucidal effect of IQC might be related to HA modulation. A recent report showed that the interaction of HA and cranberry extract results in HA inference and loss of viral infectivity [29]. IQC significantly inhibited NA activity, which is essential for viral progeny release of IAV and enhances HA-mediated viral infectivity [37]. There are many research results on the anti-neuraminidase compounds such as agathisflavone [38], isocorilagin [39], chebulinic acid [40], chebulagic acid [40], isoimperatorin [41], punicalagin [42], puerarin [43], and rosmarinic acid [44] and natural extracts such as *Epimedium koreanum* Nakai [45], Geranii Herba [46], lotus leaves [27], and *Rhus verniciflua* [47]. Collectively, IQC blocked the influenza viral binding to the cells by inhibiting hemagglutinin and exerting a virucidal effect at the early stages, and attenuated virus progeny release by repressing neuraminidase activity at the late stages during IAV infection.

## 4. Materials and Methods

### 4.1. Materials, Cells, and Viruses

Isoquercitrin with 98% purity was purchased from Wuhan ChemFaces Biochemical Co., Ltd. (Wuhan, China), and dissolved in DMSO. RAW 264.7 cells (Mouse Leukemic Monocyte Macrophage cell line; ATCC TIB-71) were grown in Le Roswell Park Memorial Institute medium (Hyclone, Logan, UT, USA) containing 10% fetal bovine serum and 100 U/mL of Penicillin and Streptomycin at 37 °C with 5% CO_2._ Green Fluorescent Protein (GFP)-tagged Influenza A/PR8/34 (PR8-GFP) and A/PR8/34 (H1N1) viruses were kindly received from Dr. Jong-Soo Lee (Chungnam National University, Daejeon, South Korea) and HBPV-VR-32 (H3N2) was purchased from the Korea Bank for Pathogenic Viruses (KBPV). The viruses were amplified in the allantoic fluid of a 10-day-old chicken embryo as described previously [48,49,50]. All virus-related experiments were performed in BL2 (Biosafety level 2).

### 4.2. Cytotoxicity Determination

To evaluate the toxicity of IQC to the cell, the CCK-8 assay was used, according to the manufacturer’s recommendation (Dojindo, Rockville, MD, USA). Briefly, RAW 264.7 cells seeded in 96 wells (1 × 10^5^ cells/well) were incubated with IQC at concentrations from 0.25 to 100 µM. At 24 h post-treatment, 10 μL of reagent was added to the plate and incubated for 1 h, and then the absorbance at 450 nm was measured using a spectrophotometer (Promega, Madison, WI, USA).

### 4.3. Anti-Influenza Viral Assay

The influenza A/PR8-GFP virus (PR8-GFP IAV) at 10 multiplicity of infection (MOI) and IQC at indicated concentrations were mixed for 1 h at 4 °C and the mixtures were added to RAW 264.7 cells for 2 h at 37 °C. After washing with PBS, the cells were further incubated for 24 h. Fluorescent microscopy or fluorescence-activated cell sorting (FACS) was used to detect GFP expression. To evaluate the inhibitory effect of IQC on the cytopathic effect (CPE) of H1N1 or H3N2 IAV, IQC with H1N1 or H3N2 at 50 MOI were placed for 1 h at 4 °C and were cotreated to RAW 264.7 cells for 2 h at 37 °C. After washing with PBS, the cells were further incubated until CPE formation. The cell viability was determined by CCK-8 assay, according to the manufacturer’s recommendation.

### 4.4. Immunofluorescence Staining

RAW 264.7 cells seeded in 12 wells (5 × 10^5^ cells/well) were incubated with a mixture of PR8-GFP IAV and 2.5 or 25 µM of IQC for 2 h at 37 °C. At 24 h post-incubation, the cells were fixed with cold absolute methanol for 10 min and 4% paraformaldehyde for 10 min. The cells were blocked with 1% BSA-PBS for 30 min and incubated with antibodies (GeneTex, Irvine, CA, USA) specific for influenza viral proteins for 1 h at room temperature. After washing with PBST (PBS with 0.05% Tween 20), the cells were incubated with Alexa Fluor 594-tagged secondary antibody (Invitrogen, Waltham, MA, USA) in PBST for 1 h in the dark. The cells were incubated with Hoechst 33342 (Invitrogen, Waltham, MA, USA) for 5 min. The images of red viral proteins and blue nuclei were captured using fluorescent microscopy.

### 4.5. Fluorescence-Activated Cell Sorting (FACS) Analysis

IQC at 0, 1, 5, 10, or 25 µM was mixed with PR8-GFP IAV for 1 h at 4 °C and the mixtures were added to the RAW 264.7 cells in 12 wells (5 × 10^5^ cells/well) for 24 h at 37 °C. The cells were washed with PBS and fixed with 4% paraformaldehyde for 10 min. The cells resuspended in PBS were analyzed using the CytoPLEX flow cell counter (Beckman Coulter Inc., Pasadena, CA, USA) to detect the levels of GFP expression.

### 4.6. Time-of-Addition Assay

The time-of-addition assay was performed on binding, entry and virucidal stages upon IAV infection. Briefly, to examine the effect of IQC on the attachment step, RAW 264.7 cells in 12 wells (5 × 10^5^ cells/well) were infected with 10 MOIPR8-GFP IAV and 25 µM IQC for 30 min at 4 °C. After washing, the cells were incubated for 24 h at 37 °C. To check the effect on the entry step, the cells were infected with 10 MOI PR8-GFP IAV for 30 min at 4 °C followed by the addition of 25 µM IQC for 30 min at 37 °C. After washing, the cells were incubated for 24 h at 37 °C. For checking the effect on the virucidal step, 10 MOI PR8-GFP IAV and 25 µM IQC were co-incubated for 30 min at 4 °C. The mixtures were added to the cells and incubated at 37 °C for 30 min. After washing, the cells were incubated for 24 h at 37 °C. The brightfield and fluorescence images of cells were obtained using fluorescence microscopy with 200× magnification. The cells were fixed with 4% paraformaldehyde for 10 min and were analyzed by FACS to compare GFP expression.

### 4.7. Hemagglutination (HA) Assay

H1N1 IAV at 10 MOI and IQC (0, 1, 5, 10, 25 µM) were mixed for 1 h at 4 °C and the mixtures were added to RAW 264.7 cells in 12 wells (5 × 10^5^ cells/well) for 2 h at 37 °C. After washing with PBS, the cells were further incubated for 24 h at 37 °C. The supernatant was harvested for the hemagglutination assay. Briefly, each supernatant was serially two-fold diluted and added to a round-bottomed 96-well plate. The blank medium was used as a negative control. Each sample (100 µL) was mixed with an equal volume of 1% chicken red blood cells (RBCs) (Innovative Research, Inc., Southfield, MI, USA) in PBS, and incubated for 1 h at room temperature. RBCs in negative control exhibited agglutination and RBCs in the virus-infected well were lysed without agglutination. HA titers were calculated as HA unit/100 µL in comparison with values of the virus control treatment. HA units were determined as the point at which hemolysis occurs.

### 4.8. Neuraminidase (NA) Inhibition Assay

A neuraminidase (NA) inhibition assay was performed using the NA-Fluor influenza Neuraminidase Assay Kit (Life Technologies, Carlsbad, CA, USA) according to the manufacturer’s instructions. IQC was serially 2-fold diluted from 100 μM to 1.56 μM in 96 well black plates. As a positive control, oseltamivir carboxylate (Aobious Inc, MA, USA), a specific NA inhibitor, was used from 10 μM to 0.0001 μM. H1N1 or H3N2 (2 × 10^6^ PFU) IAV was added to each well and incubated for 30 min at 37 °C. The mixtures were incubated with 200 µM NA-Fluor Substrate for 1 h for 37 °C and terminated using NA-Fluor stop solution. The reaction was monitored by a fluorescence spectrometer (Promega, Madison, WI, USA) with an excitation wavelength of 365 nm and an emission wavelength of 445 nm.

## 5. Conclusions

Isoquercitrin, an ingredient abundantly found in natural products, prevented IAV binding and entry into the cells and exhibited a strong virucidal effect at an early stage upon IAV infection. Additionally, isoquercitrin inhibited viral progeny release via blocking neuraminidase activity. Our results elucidated the underlying mechanism of the antiviral efficacy of isoquercitrin against IAV infection and suggested the potential for development as an antiviral agent.

## Data Availability

The data in this study are available on request from the corresponding author.
